# Biosynthesis and Biodegradation—Eco-Concept for Polymer Materials

**DOI:** 10.3390/ijms25052674

**Published:** 2024-02-26

**Authors:** Joanna Rydz, Wanda Sikorska, Marta Musioł

**Affiliations:** Centre of Polymer and Carbon Materials Polish Academy of Sciences, M. Curie-Skłodowska 34, 41-819 Zabrze, Poland; wsikorska@cmpw-pan.pl (W.S.); mmusiol@cmpw-pan.pl (M.M.)

Polymers have become essential for various aspects of modern life, including packaging, transportation, and electronics. However, the widespread use and disposal of these polymers have led to significant environmental issues. They contribute to pollution in oceans, harm wildlife, and take centuries to decompose. Therefore, it is crucial to find sustainable alternatives and improve waste management systems to mitigate these negative consequences and minimize the impact on the environment [1].

The need to find alternative materials to conventional plastics that are environmentally friendly, green, and sustainable has driven significant growth in research focusing on the design, characterization, production, and development of such materials. These include resource-efficient materials that aim to improve efficiency and reduce costs. This field of research is vital for addressing the pressing environmental challenges we face and creating a more sustainable future [2,3]. The European Union strategy identifies the most important actions that national and regional authorities as well as industry should take. These actions aim to strengthen the single market, promote investment and innovation, address social and environmental challenges, and ensure a competitive and sustainable economy [4]. The current advanced production chain combines various intermediate links, such as raw material suppliers, producers of high-value polymer materials, and recyclers. The integration of these intermediate links in the production chain is crucial for ensuring efficiency and sustainability. Recyclers play a significant role in closing the loop by collecting and reusing any leftover materials, reducing waste and environmental impact. This interconnectedness promotes resource conservation and a circular economy [5]. Both biosynthesis, such as the production of complex chemicals from simpler precursors as a result of enzymatic reactions by a living organism, and biodegradation, such as the decomposition of substances catalyzed by enzymes in vivo, fit into the assumptions of green chemistry, which uses a set of principles that limit or eliminate the use or generation of hazardous substances when designing, manufacturing, and using chemical products [6]. Green chemistry aims to minimize or eliminate the use of hazardous substances in chemical processes, and both biosynthesis and biodegradation offer sustainable and environmentally-friendly alternatives to traditional chemical production and waste management methods [7].

In response to the need for more sustainable solutions, research is leading to the development of functional materials that not only perform efficiently, but also have a positive impact on the environment. (Bio)degradable polymers, which can replace non-degradable conventional plastics, are particularly significant due to their potential in various applications, their contribution to environmental preservation, and their support for long-term sustainable development. (Bio)degradable polymers offer a promising solution to reduce environmental pollution and waste accumulation caused by conventional non-biodegradable materials. In particular, the use of (bio)degradable polymers in agriculture can improve soil health and reduce plastic waste in the ecosystem, while in food and chemistry industries, as well as medicine, it can lead to the development of more sustainable products with improved performance and functionality. Blends of poly(lactic acid) (PLA) with poly(propylene carbonate) (PPC) have been shown to have potential in various applications such as packaging materials, biomedical devices, and eco-friendly coatings. The mechanical, thermal, and biodegradable properties of these polymer blends make them attractive alternatives to traditional petroleum-based plastics. However, further research is still needed to optimize the blend compositions and processing conditions to fully exploit their potential [8,9,10,11].

One way to optimize the properties is to obtain a liquid crystal phase in a (bio)degradable polymer. The non-mesogenic thermoplastic (bio)degradable polymers can undergo a phase transition to exhibit liquid crystal properties under specific conditions, including temperature regulation, heating time, pressure force, and the addition of fine powder. By controlling these factors, a chiral nematic mesophase can be formed in the polymer. The ordered fluid phases of liquid crystals have unique properties that make them valuable for various applications in fields like medicine and packaging. These include their use as precursors for high-performance polymer films, fibers, and injection-molded items. In the medical industry, liquid crystals are utilized in artificial irises and blood sensors, while in the packaging industry, they are used in smart packaging as smart displays. These applications highlight the versatile and valuable nature of liquid crystals in various industries. When these properties are combined with biodegradability, it is possible to obtain unique environmentally friendly polymers [12,13].

Natural polymers derived from proteins or polysaccharides have also a wide range of applications, including in tissue engineering, drug delivery, wound healing, and food science. The natural polymers offer advantages such as excellent biocompatibility, biodegradability, waste reduction, and sustainable sourcing, making them attractive for various industries. Tissue engineering and regenerative medicine utilize additive technologies and hydrogels to create structures that mimic the extracellular matrix and provide mechanical support to cells. These advancements aim to repair damaged organs and tissues, offering potential solutions for diseases or injuries [14]. 

Collagen-based polymers and their blends are highly appealing for the development of new materials. Their exceptional biocompatibility, physico-mechanical properties, and durability make them a versatile option for various applications. These materials have the potential to be used in the medical field for tissue engineering and drug delivery, as well as in other industries such as cosmetics and food packaging. The research and development of collagen-based polymers open up opportunities for the creation of innovative materials that can meet the growing demands of a wide range of industries. Leather is a versatile material that has been used for centuries. Manufacturers are exploring innovative techniques and materials to reduce the environmental impact of leather production, such as using vegetable-based tanning agents, recycling water, and chemicals, and exploring alternative sources of leather. By focusing on sustainable practices, the leather industry aims to meet the increasing demand for eco-friendly products while maintaining high quality and cost-effectiveness. The unique mixture of a modified natural biopolymer made from animal rawhide and the first synthetic collagen-based polymer with its natural properties, such as durability, flexibility, and breathability, makes this new material ideal for various applications, from clothing and accessories to furniture and automotive upholstery [15,16,17].

Gelatin methacryloyl is a hydrogel that is gaining popularity due to its unique ability to modify its mechanical properties by adjusting factors like functionalization, concentration, and photo-crosslinking parameters. This versatility makes it an attractive material for various applications in tissue engineering and drug delivery [18].

On the other hand, nanotechnology offers a solution for protecting molecules with physico-chemical limitations through techniques like nano-encapsulation. By utilizing (bio)degradable polymers, nanotechnology can minimize volatilization and enhance the overall effectiveness of these molecules. Nanotechnology offers potential benefits in many areas of life, including the development of repellents against insect-borne diseases. By encapsulating active compounds, nanotechnology can protect them from degradation, enhance effectiveness, and reduce toxicity. This technology can enable controlled release of the active compound, thereby minimizing adverse reactions, making it a promising tool to protect people and the environment [19,20].

The activated sludge process, which is currently the most widespread method of wastewater treatment, is irrevocably linked to a high production of sludge that cannot be directly and safely neutralized in the environment due to its quality and characteristics. Efforts to find sustainable and efficient solutions for sludge management in wastewater treatment are crucial. Developing universal and cost-effective methods for neutralizing sludge is essential in order to minimize environmental impact and support the continuous expansion of wastewater treatment systems. Efficient management of wastewater sludge is crucial to minimizing its environmental impact. One promising solution is the use of solid CO_2_ in the pretreatment and management of sewage sludge. This approach effectively addresses previous challenges, such as the high cost of production, by offering a cost-effective alternative for solidified CO_2_ generation and application [21,22].

Aerobic granular sludge is a type of organic waste that has recently gained attention in the municipal sector. Its unique characteristics suggest that it could be a viable material to use as a substrate for producing bio-hydrogen. Dark fermentation, a process in which organic materials are broken down by bacteria in the absence of light, has the potential to address some of the technological challenges associated with hydrogen fermentation. Although it is considered a promising method, the bioconversion of organic waste into bio-hydrogen via dark fermentation has many disadvantages, such as low hydrogen yield, slow fermentation rate sensitivity to environmental factors, and the production of by-products such as volatile fatty acids and alcohols that can inhibit the fermentation process. Additionally, the process requires strict control over operating conditions, making it less economically viable compared to other renewable energy sources. By combining dark fermentation with fermentation of organic acids to produce methane, biohythane production can be achieved, reducing the dependency on pure hydrogen production. This approach can help to overcome issues such as low hydrogen yield, sensitivity to impurities, and the requirement for strict operating conditions, making it a promising alternative for hydrogen fermentation [23,24].

The aim of this Special Issue is to provide a contemporary overview of the latest developments and current trends in the field of materials, in particular, the latest breakthroughs and approaches in the science of bio-based and/or (bio)degradable polymers leading to the development of new-generation, resource-efficient, environmentally friendly, and sustainable polymer materials as a new eco-concept of polymer materials for the circular economy.

## Data Availability

Not applicable.
